# How do we measure attention? Using factor analysis to establish construct validity of neuropsychological tests

**DOI:** 10.1186/s41235-021-00313-1

**Published:** 2021-07-22

**Authors:** Melissa Treviño, Xiaoshu Zhu, Yi Yi Lu, Luke S. Scheuer, Eliza Passell, Grace C. Huang, Laura T. Germine, Todd S. Horowitz

**Affiliations:** 1 Basic Biobehavioral and Psychological Sciences Branch, National Cancer Institute, 9609 Medical Center Drive, Rockville, MD 20850 USA; 2 Westat, 1600 Research Boulevard, Rockville, USA; 3 Institute for Technology in Psychiatry, McLean Hospital, Belmont, USA; 4 Department of Psychiatry, Harvard Medical School, Cambridge, USA

## Abstract

We investigated whether standardized neuropsychological tests and experimental cognitive paradigms measure the same cognitive faculties. Specifically, do neuropsychological tests commonly used to assess attention measure the same construct as attention paradigms used in cognitive psychology and neuroscience? We built on the “general attention factor”, comprising several widely used experimental paradigms (Huang et al., 2012). Participants (*n* = 636) completed an on-line battery (TestMyBrain.org) of six experimental tests [Multiple Object Tracking, Flanker Interference, Visual Working Memory, Approximate Number Sense, Spatial Configuration Visual Search, and Gradual Onset Continuous Performance Task (Grad CPT)] and eight neuropsychological tests [Trail Making Test versions A & B (TMT-A, TMT-B), Digit Symbol Coding, Forward and Backward Digit Span, Letter Cancellation, Spatial Span, and Arithmetic]. Exploratory factor analysis in a subset of 357 participants identified a five-factor structure: (1) attentional capacity (Multiple Object Tracking, Visual Working Memory, Digit Symbol Coding, Spatial Span), (2) search (Visual Search, TMT-A, TMT-B, Letter Cancellation); (3) Digit Span; (4) Arithmetic; and (5) Sustained Attention (GradCPT). Confirmatory analysis in 279 held-out participants showed that this model fit better than competing models. A hierarchical model where a general cognitive factor was imposed above the five specific factors fit as well as the model without the general factor. We conclude that Digit Span and Arithmetic tests should not be classified as attention tests. Digit Symbol Coding and Spatial Span tap attentional capacity, while TMT-A, TMT-B, and Letter Cancellation tap search (or attention-shifting) ability. These five tests can be classified as attention tests.

## Significance statement

Assessment of cognitive function in clinical populations, for both clinical and research purposes, is primarily based on standardized neuropsychological testing. However, this approach is limited as a clinical research tool due to two major issues: sensitivity and construct validity. Deriving new measures based on contemporary work in cognitive psychology and cognitive neuroscience could help to solve these problems. However, we do not understand the relationship between existing neuropsychological tests and widely used cognitive paradigms. The goal of this paper is to begin to address this problem, using factor analysis tools to map the relationships, specifically in the domain of attention. Our results should provide guidance for which neuropsychological tests should be classified as attention tests, and hopefully provide inspiration for the development of new clinical assessments based on experimental attention paradigms. Furthermore, we hope we have provided a template for other researchers to explore the connections between cognitive paradigms and neuropsychological tests in domains beyond attention. By bringing these fields closer together, we can improve our scientific understanding of cognition, and ultimately improve the welfare of people who suffer from cognitive disorders and deficits.

## Introduction

Assessing cognitive functioning across the gamut of health and mental health conditions has traditionally relied on standardized neuropsychological test batteries (Foti et al., 2017; Helmstaedter et al., 2003; Meade et al., 2018; Vives et al., 2015). However, this approach may be reaching its limits as a clinical research tool in many fields, due to two major issues: sensitivity and construct validity (Bilder & Reise, 2019; Horowitz et al., 2018; Howieson, 2019; Kessels, 2019; Marcopulos & Łojek, 2019; Parsons & Duffield, 2019). We and others have proposed that deriving new measures based on contemporary work in cognitive psychology and cognitive neuroscience could help to solve these problems (Carter & Barch, 2007; Horowitz et al., 2018). However, we currently do not understand the relationship between existing neuropsychological tests and widely used cognitive paradigms. The goal of this paper is to begin to address this problem, using factor analysis tools to map the relationships. Specifically, we will address the attention domain, which was the most frequently assessed cognitive domain in our survey of cancer-related cognitive impairment studies (Horowitz et al., 2019).

Many neuropsychological tests were originally designed to diagnose severe cognitive difficulties (e.g., resulting from stroke). As a result, they tend to lack sensitivity to the less severe, and often more diffuse cognitive difficulties encountered by many clinical populations (Nelson & Suls, 2013). This insensitivity may contribute to the widely observed lack of correlation between objective neuropsychological tests and patients’ subjective reports of their own cognitive problems (Jenkins et al., 2006; Srisurapanont et al., 2017).

Neuropsychological tests tend to be developed from a practical rather than a theoretical standpoint, and often tap multiple cognitive abilities in a single test (Sohlberg & Mateer, 1989). This means that it is often difficult to know exactly what cognitive faculties are being measured by a given test (Kessels, 2019; Schmidt et al., 1994). The Digit Symbol Coding test, for example, is a widely used neuropsychological test that is variously held to measure attention, psychomotor speed, working memory, processing speed, and executive function (Horowitz et al., 2019). In the clinical setting, this lack of specificity can be an advantage. If a patient has cognitive problems, they are likely to show up on the Digit Symbol Coding test. However, the downside is that it is very difficult to pin down which cognitive faculties are affected (Jaeger, 2018). For research purposes, this construct validity problem is a major limitation (McFall, 2005; McFall & Townsend, 1998), and poses a general challenge to integrating neuropsychological research with cognitive neuroscience (Horowitz et al., 2019).

In contrast to the neuropsychological tradition, experimental paradigms (“paradigms” rather than “tests”, because there is no standard version; Kessels, 2019) in basic cognitive psychology and cognitive neuroscience are explicitly created to test theoretical models of specific cognitive functions and operations. Experimental paradigms often have internal manipulations that allow for separations of subcomponent processes. Consider the visual search paradigm, in which observers search through N items to find a target (e.g., search for the T among Ls). Instead of looking at the overall response time, the experimenter computes the slope of the regression line for response time as a function of N to yield a pure search rate, independent of perceptual, response, and motor stages (Sternberg, 1966). Similarly, in the Eriksen flanker paradigm (Eriksen & Eriksen, 1974) participants see three letters, and are asked to give one response if the central letter belongs to one category (e.g., X or T) and another response if it belongs to another (e.g., O or C). If the two flanking letters come from the same category as the target (e.g., X T X, compatible trial), responses are typically faster than when they come from different categories (e.g., X C X, incompatible). The primary dependent measure is again not overall response time, but the incompatible-compatible difference score, which provides a measure of the strength of interference from incompatible responses. This sort of logic is rare in neuropsychological tests, perhaps in part because they have been largely administered, even now, as paper-and-pencil tests. Consider the Trail Making Test (Partington & Leiter, 1949), in which participants are asked to connect a sequence of digits in order (1, 2, 3, etc., version A) or alternate between digits and letters (1, A, 2, B, etc., version B). The score for each version is overall completion time. This score conflates search ability, motor function, and executive function into a single score. As with the Digit Symbol Coding test, this makes the Trail Making Test sensitive to a wide range of deficits, which contributes to its popularity (along with the fact that it is not proprietary), while making the results difficult to interpret (Kessels, 2019) though several groups have attempted to decompose Trail Making Test performance (Crowe, 1998; Misdraji & Gass, 2010; Salthouse, 2011). Interestingly, the Trail Making Test does produce a difference score: the difference between versions A and B is held to measure target-switching or executive function (Crowe, 1998; Sánchez-Cubillo, 2009). However, this difference score is rarely reported in the scientific literature (e.g., in the cancer-related cognitive impairment field, see Horowitz et al., 2019).

One way to bridge this gap is to take experimental paradigms and adapt them to the demands of clinical research and practice. Several recent ventures in this direction have been based on Posner and Petersen’s (1990) influential Attentional Network theory, which proposed that attention is divided into three separate functional brain networks. The alerting network is defined as maintaining a vigilant and alert state, the orienting network involves directing attention in space, and the executive control is responsible for resolving conflict between responses (MacLeod et al., 2010). Based on this conceptualization of attention, Fan et al. (2002) developed the Attentional Network Test, which combines a flanker task (with incongruent, congruent, and neutral trials) and a cued reaction time task (with central, double, spatial, and no cue trials). The difference between trials with congruent and incongruent flankers measures executive control, the difference between central and spatial cues measures orienting, and the difference between double and no-cue trials measures orienting. The Dalhousie Computerized Attention Battery (Jones et al., 2016) was also designed to measure the alerting, orienting, and executive control networks, using a battery of eight tasks adapted from the experimental literature. Simple and choice reaction time tasks measure alerting, visual search measures orienting, while a go/no go task, a dual task, a flanker task, and item and location working memory tasks measure executive control. The NIH Toolbox (https://www.healthmeasures.net/explore-measurement-systems/nih-toolbox) is a comprehensive set of computerized tests designed to measure cognitive, emotional, sensory, and motor functions. The NIH Toolbox Cognition Battery, part of the NIH Toolbox initiative, uses a version of the flanker test derived from the ANT to measure inhibitory control (Zelazo et al., 2013).

The ANT has been adopted fairly widely. A review by Arora, Lawrence, and Klein (Arora et al., 2020) found 889 studies using the ANT through 2019. Similarly, the paper describing the NIH Toolbox Cognition Battery Executive Function and Attention components has been cited in 244 papers as of early 2021. The Dalhousie battery has not had much time to gain traction; we could only find two non-methodological papers that had cited it (Cunningham et al., 2018; Sardiwalla et al., 2019). However, none of the three batteries showed up in our survey of meta-analyses of cancer-related cognitive impairment (Horowitz et al., 2019), or in a survey of practicing clinical neuropsychologists (Rabin et al., 2016).

We propose that factor analysis can serve as a useful tool to help establish construct validity. By mapping the relationships between neuropsychological tests and experimental paradigms, we can gain a better understanding of how neuropsychological tests relate to contemporary theories in cognitive psychology and cognitive neuroscience. Our approach is to run a set of participants on a battery composed of experimental cognitive paradigms from the attention literature and neuropsychological tests commonly used to measure attention in clinical populations, and use factor analysis to see whether the neuropsychological tests load on the same factor as the experimental paradigms.

Factor analysis is widely used in the development and validation of neuropsychological test batteries, but typically this is only done to understand the factor structure within a battery (e.g., Jones et al., 2015; Price et al., 2002). Two studies have used factor analysis to understand the relationships among neuropsychological tests commonly used to assess attention. Mirsky et al. (1991) found that many tests loaded onto a “perceptual-motor speed” factor (including Stroop, the Trail Making Test, Digit Symbol Coding, and Digit Cancellation), a “numerical-mnemonic” factor (Digit Span and Arithmetic) a “vigilance” factor (Continuous Performance Test) and a “flexibility” factor (Wisconsin Card Sorting Test). Schmidt et al. (1994) found separate factors for “scanning” (Stroop, TMT-A & -B, Digit Symbol Coding) and “spanning” (Visual Span, Digit Span Forwards, Digit Span Backwards).

In studies of intelligence, the Cattell–Horn–Carroll taxonomy (McGrew, 2009) is an influential factor-analytic scheme for organizing tests into domains (termed “broad abilities). While the taxonomy was developed on intelligence batteries, there is a certain amount of overlap with neuropsychological testing, where batteries (or tests from batteries) such as the Wechsler Adult Intelligence Scale often serve clinical purposes. Recent meta-factor-analytic work from Jewsbury et al. (2017) and Agelink van Rentergem et al. (2020) show that the Cattell–Horn–Carroll framework also fits well to data from neuropsychological batteries.

Studies that have tried to map clinical neuropsychological tests to laboratory or experimental paradigms are rare. In the working memory domain, Shelton et al. (2009) showed that clinical tests from the Wechsler batteries (Wechsler Adult Intelligence Scale III and Wechsler Memory Scale) correlated poorly with a factor defined by laboratory paradigms such as operation span (OSPAN, Turner & Engle, 1989), while the laboratory paradigms better predicted fluid intelligence. To our knowledge, no one has used factor analysis to study the relationship between neuropsychological attention tests and experimental attention paradigms.

As an object of study, attention lacks the sort of strong criterion construct that fluid intelligence presents for working memory (Shelton et al., 2009). However, a study from Huang et al. (2012) provides a useful starting point. Huang et al. tested 257 participants on 17 different experimental paradigms drawn from the attention literature, including nine “primary” and eight “secondary” paradigms, selected for their theoretical relevance as attention measures (Bundesen, 1990; Desimone & Duncan, 1995; Huang & Pashler, 2007; Posner & Petersen, 1990; Treisman & Gelade, 1980). In a principal components analysis, all nine primary paradigms loaded strongly on a single general attention factor, which Huang et al. termed *a*, with analogy to *g*, the general intelligence factor. The *a* factor accounted for 35% of total task variance, while 65% of the variance was explained by task-specific mechanisms. This result suggests that there is a single underlying attention mechanism that can be measured with one of the nine primary paradigms. To be precise, *a* should be regarded as a selective attention factor, as there were no sustained attention paradigms in Huang et al.’s battery.

In contrast, a study from Skogsberg et al. (2015), who tested 222 participants on 11 tasks, concluded that attention measures could be meaningfully divided into four clusters: spatiotemporal attention, global attention, transient attention, and sustained attention. Unfortunately, the set of attention tests analyzed by Skogsberg et al. has very little overlap with the Huang et al. (2012) set; only Multiple Object Tracking was included in both analyses. It is possible that Huang et al.’s *a* is a subset of the four clusters identified by Skogsberg et al. We return to this topic in the discussion.

The plan for the current study was to determine whether neuropsychological tasks used to measure attention would load on to the same factor(s) as tests derived from experimental cognitive paradigms. We chose to design our study around Huang et al. (2012)’s *a* factor. By using a single factor that would correlate with multiple tests, we thought to achieve more power while simplifying the interpretation of the data.

We designed a battery composed of six experimental tests, and eight neuropsychological tests. The experimental tests included five selective attention paradigms, and one sustained attention test. The five selective attention tests (multiple object tracking, spatial configuration visual search, visual working memory, approximate number sense, and flanker interference) were selected to represent the paradigms that had the strongest correlations (i.e., 0.56–0.67) to the *a* factor in Huang et al.’s (2012) study, were widely used in experimental, cognitive psychology, and had strong theoretical justifications as attention measures. It is important to note that our tests were not identical to those used by Huang et al. This project was not an attempt to replicate Huang et al., but to build on the concept of the general attention factor. In some cases (multiple object tracking, visual working memory, approximate number sense, and flanker interference), we opted to use tasks that were already available on the TestMyBrain platform.

The multiple object tracking (MOT) task corresponds to the Tracking paradigm in Huang et al.’s (2012) battery. In MOT, participants need to remember and track a set of targets among a larger set of identical distractors. This requires selective attention to track targets while excluding distractors. Selective attention is central to successful performance in these tasks (Holcombe et al., 2014; Vul et al., 2009), and the paradigm has been a useful proving ground for models of attention (Cavanagh & Alvarez, 2005; Pylyshyn & Storm, 1988; Scholl, 2009). A version of the MOT was included in the Skogsberg et al. (2015) battery. Our MOT task asked participants to track 3–5 out of 10 disks, while Huang et al.’s Tracking paradigm had participants track 4 out of 8 disks.

Visual search has played a central role in attentional theory for decades (Schneider & Shiffrin, 1977; Treisman & Gelade, 1980). Spatial configuration search, where targets are distinguished from distractors only by the internal arrangement of components, is widely held to index serial shifts of covert attention (Bricolo et al., 2002; Wolfe, 2021; Woodman & Luck, 1999). Here we employed the widely used spatial configuration search for T-shaped targets among L-shaped distractors. This corresponds to Huang et al.’s (2012) Configuration Search task for squares that were white-above-black among black-above-white.

Visual working memory (VWM) may seem like an odd choice to measure attention, especially when we are trying to distinguish between attention and working memory functions. However, VWM is a distinct, modality-specific memory store (Fougnie & Marois, 2011), and is tightly linked to selective attention, in that both encoding (Emrich et al., 2017; Praß & de Haan, 2019) and maintenance in VWM require visual attention (Makovski et al., 2008; Roper & Vecera, 2014; Sandry & Ricker, 2020). Huang et al. (2012) used a Visual Short-Term Memory task requiring participants to memorize an array of six colors and then recreate this array from memory after it offset (i.e., full report paradigm). However, it is much more common to use a change-detection paradigm to measure visual short-term or working memory capacity (Luck & Vogel, 1997; Pashler, 1988). In a change detection paradigm, the whole array is presented at recall, and the participant has to indicate whether or not any element has changed. This approach is more time-efficient, and also avoids the complication of changes in the state of the memory during an extended report process (Peters et al., 2018). Accordingly, we measured Visual Working Memory (VWM) in a change-detection paradigm where participants had to memorize four shapes and report whether one of them changed after a brief delay.

Enumeration and numerosity perception are also tightly linked to selective attention. Specifically, enumeration can be described as an attentional individuation mechanism (Mazza & Caramazza, 2015). Whether we perceive precise counts or estimates depends on whether attention is focused or distributed (Chong & Evans, 2011). To measure numerosity perception, Huang et al. (2012) employed a Counting task that required participants to report whether the number of dots on the screen was even or odd. We opted for the Approximate Number Sense (ANS, Halberda et al., 2012) task that required participants to indicate whether there were more blue dots than yellow dots or vice versa. The ANS task is more strongly linked to attention, since it is a selective enumeration task, requiring participants to filter out irrelevant items.

The final paradigm was response selection, a form of internal attention (Chun et al., 2011) involving selection between competing actions. Huang et al. (2012)’s Response Selection task was a 4-alternative forced-choice response to the color of a ball. We chose the Flanker Interference task (Eriksen & Eriksen, 1974), which requires participants to respond to a central target in the presence of irrelevant flanking stimuli that could be congruent or incongruent with the correct response. This choice was made partly for theoretical reasons, in that the requirement to filter out distraction makes the Flanker Interference task more of a selective attention task than a forced-choice response time task. Additionally, the Flanker Interference task is more widely used, both in experimental cognitive psychology and in neuropsychology. The Attentional Network Task (Fan et al., 2002), the Dalhousie Computerized Attention Battery, and the NIH Toolbox Cognition Battery executive function and attention sub-battery (Zelazo et al., 2013) all include a Flanker Interference component.

Finally, we also included a sustained attention test, the Gradual Onset Continuous Performance Test (gradCPT). The gradCPT is similar to continuous performance tasks that require frequent responses, such as the Sustained Attention to Response Task (Robertson et al., 1997), except that the gradCPT uses gradual transitions between stimuli, rather than abrupt onsets that may capture attention (Yantis & Jonides, 1990) and thus reduce sensitivity to vigilance decrements (Rosenberg et al., 2013). The gradCPT has been demonstrated to be sensitive to individual differences (Fortenbaugh et al., 2015; Rosenberg et al., 2013).

In contrast, the eight neuropsychological tests (Trail Making Test versions A & B (TMT-A, TMT-B), Digit Symbol Coding, Forward and Backward Digit Span, Letter Cancellation, Spatial Span, and Arithmetic) were *not* chosen for their theoretical or empirical links to attention. We selected the tests most frequently used to measure attention in our review of the literature on cancer-related cognitive impairment in cancer survivors (Horowitz et al., 2019). Digit span, arithmetic, letter cancellation, and the Trail Making Test were also among the most frequently used tests for “attention, concentration, and working memory” in a survey of the membership lists of the National Academy of Neuropsychology and the International Neuropsychological Society (Rabin et al., 2016), so we believe that this usage is typical of neuropsychological practice.

Historically, the neuropsychological tests used to measure attention have not been grounded in attentional theory (Mirsky et al., 1991; Schmidt et al., 1994). Tests such as Digit Span (measuring the number of digits participants can recall) and Arithmetic (ability to solve mathematical word problems) would seem to have little relationship to attention, since they do not require any sort of selection or filtering. Indeed, in our database of cancer-related cognitive impairment studies (Horowitz et al., 2019), these tests are also frequently classified under working memory, rather than attention. Then again, given that *visual* working memory and numerosity perception tests do seem to be linked to attention, we should not rule out these tests as attention measures out of hand. The Trail Making and Letter Cancellation tests closely resemble the visual search paradigm. However, as noted above, it is difficult to parse out the search component from motor factors or ability to alternate sequences (in the TMT-B). The Digit Symbol Coding test, in which participants are given a symbol-number key and asked to match a presented symbol to its corresponding number within an allowed time, similarly seems to tap into a number of cognitive domains, including visual search. Spatial Span, a visuospatial analog of the Digit Span tests, may be related to multitarget spatial attention tasks such as MOT (Trick et al., 2012). Notably, the Spatial Span test is alone among the neuropsychology tests we used in that it is never classified as anything other than an attention test in our dataset (Horowitz et al., 2019).

We hypothesized that the five selective attention paradigms would load on a common factor, *a*. We included the sustained attention paradigm on a hunch that some of the neuropsychological tests were better at predicting vigilance than selection. The key research question was which of the neuropsychological tests, if any, would also load on the *a* factor.

## Method

### Participants

#### Sample size

Recommendations for determining the minimal sample size for factor analysis have been diverse and often contradictory (MacCallum et al., 1999; Preacher & MacCallum, 2002), though Thompson (2004) suggested that a sample size of 300 is generally considered sufficient. That said, there are some basic guiding principles to take into account when determining sample size, including the strength of the relationship between variables and factors (measured by level of communality), the number of factors, and the number of variables per factor. Smaller sample sizes are required when the communality is higher and the variable-to-factor ratio is higher (Mundfrom et al., 2005).

The current study assumes two general factors underlying fourteen measures, a variable to factor ratio of 7:1. According to the Monte Carlo study conducted by MacCallum et al. (1999), a sample size of 200 can achieve an excellent recovery when the communality is low, if the variable-to-factor ratio is 20:3 or greater. Inter-correlations between measures that are assumed to load on to one factor are expected to be significant. We were able to estimate pairwise correlations among six of our fourteen measures from previous studies in the TestMyBrain database. Some of these correlations were lower than 0.15. To detect a correlation of 0.15 with power of 0.80, the required sample size is 350. We therefore aimed to obtain 350 participants.

#### Recruitment

We recruited participants from three sources: Visitors to TestMyBrain.org, an online cognitive testing platform, who clicked on a link titled “Cancer and Cognition”; Constant Contact (secure email marketing service) email blasts to participants signed up for the Citizen Science 4 Brain Health community; advertisements shared with cancer-focused mailing lists including the National Cancer Institute’s email list, Cancer Carepoint, Gryt Health, Cancer Survivorship, and American Cancer Society’s network. Recruitment invitations included a brief study description and informed participants that they would get feedback on their scores after completing the study.

#### Inclusion/exclusion criteria

We included only participants between the ages of 18 and 89 at the time of recruitment. We excluded participants who had a disability that substantially interfered with their ability to complete neurocognitive tests and/or were a current resident of the European Union or European Economic Area. To satisfy the General Data Protection Regulation, the consent form stated that residents from the European Union or European Economic Area were not allowed to participate. Additionally, since we are interested in determining whether our results will generalize to a population of cancer survivors, the exclusion criteria for the current study included a current or past diagnosis of cancer. Data from participants with a current or past diagnosis of cancer will be reported in a separate paper.

### Procedure

Participants began the study by clicking on a link that took them to a study information/online consent form. Once they had read the information and agreed to the consent form, they were then directed to the study and given a link with a coded ID that they could use to access the study at a future time, if needed. Participants were not required to complete the study in a single session. Coded IDs were not linked with email addresses or other personal identifying information.

When participants first clicked on the link to the study, they were taken to a page that asked for their age and the type of device they were using. Participants who reported an age younger than 18 or older than 89 were not allowed to continue the study. Next, participants were informed that they would receive the results of their assessments once they completed the study. They were then asked a series of questions to ascertain their demographics (age, gender, race, ethnicity, educational background) and cancer history (diagnosis and treatment), if any.

Once they had answered these questions, they began the cognitive testing battery. The battery took around 90 min to complete. There were four possible testing orders, counterbalanced across participants. Time of completion of each test was captured in the data to allow for any performance differences that arose from participation across multiple sessions to be modeled in our data analysis.

After participants completed all measures, they were given a debriefing questionnaire which asked about any challenges or technical difficulties they may have experienced during testing. Finally, participants were presented with results of their assessment, based on comparing their scores to the scores of participants from the TestMyBrain normative database, along with links to resources to address any concerns they might have about their cognitive health, or any questions about cancer and cognition.

### Measures

Measures were divided into those that were adapted from traditional tests of neuropsychological functioning and paradigms adapted from the experimental literature.

#### Neuropsychological tests

##### Arithmetic

The Arithmetic test required participants to solve a series of 20 arithmetic word problems of increasing difficulty (e.g., “How many hours will it take to walk 24 miles at a rate of 3 miles per hour?”). For each trial, regardless of difficulty, the participant earned 1 point for a correct answer and 2 points for a correct answer given within 10 s. The primary outcome variable was total points earned across the test. The first 5 questions had a 15 s time limit, which increased to 30 s for questions 6–10, 60 s for questions 11–19, and 120 s for the final question. This test was modeled after the arithmetic test of the Wechsler Adult Intelligence Scale, 3rd Edition (Wechsler, 1997).

##### Trail making test, parts A and B

Participants were presented with a display of 25 circled alphanumeric characters. The task was to draw lines, on the device screen, connecting the circles in the proper order. In Part A, the circles contained digits that had to be connected in ascending numerical order (i.e., “1”, “2”, “3”… “25”) starting with “1”. In Part B, the circles contained both digits and letters that had to be connected in ascending numerical and alphabetical order, switching between digits and letters (i.e., “1”, “A”, “2”, “B”, “3”, “C”… “13”). Depending on the device (i.e., laptop/desktop or tablet/smartphone), participants could use either their mouse or their finger to connect the circles. As these two response types have different motor demands, we also corrected for device/input type in all analyses (Germine et al., 2019). The primary outcome variable for each part was the total time to connect all circles. This test was modeled after the classic Trail Making Test (Partington & Leiter, 1949; Reitan, 1971), adapted for digital administration.

##### Digit span, forward and backward

These tests required participants to recall sequences of digits of increasing length. Sequences were presented serially, one digit at a time. Each digit was presented at the center of the screen for 1 s. Then, the final digit was removed and the participant had to type in the sequence. For the Forward Digit Span, the sequence had to be typed in as it was originally presented. For the Backward Digit Span, the sequence had to be typed in reverse order. They had 4 s to respond. The test began with 2 digit sequences. There were two trials for each sequence length. If the participant was correct on at least one of the two trials, the sequence length was increased for the next trial, up to 11 digits. The test ended when the participant missed two trials of the same length. The primary outcome measure for both tests was the length of the longest sequence where participants got at least one of two trials right. These tests were modeled after the Digit Span Forward and Digit Span Backward tests from the Wechsler Adult Intelligence Scale, 3rd Edition (Wechsler, 1997). For details, see Hartshorne and Germine (2015).

##### Digit symbol coding

In this test, participants had to match a target symbol with its corresponding digit. On each trial, participants were shown a novel symbol at the top of the screen. Underneath the symbol was a key mapping each of 9 novel symbols to one of the digits 1–3. The participant had to type in the digit that corresponded to the target symbol. Participants had 90 s to complete as many trials as possible. The primary outcome measure was the number of correct trials. This test was modeled after the Digit Symbol Coding test from the Wechsler Adult Intelligence Scale, 3rd Edition (Wechsler, 1997). For details, see Hartshorne and Germine (2015).

##### Letter cancellation

The Letter Cancellation test was a search test where the target was a conjunction of the letter “d” and two line segments, and the distractors were the letter “d” with one, three, or four line segments, and the letter “p” with one to four line segments. The line segments could be above the letter, below the letter, or both. There were 57 letter + line segment items, arranged in a 6 × 10 grid with the rightmost three spaces on the bottom row blank. The task was to click on all instances of the target. Whenever the participant correctly clicked on a target, it turned red. There were 14 trials. Trials timed out after 20 s. The outcome variable was the total number of targets correctly detected. This test was modeled after the D2 Test (Brickenkamp & Zillmer, 1998).

##### Spatial span

In this test, participants saw an array of 16 circles, arranged in concentric groups of four around a central point. Circles were progressively larger moving out from the center. At the start of a trial, all of the circles flashed briefly. Then, a sequence of circles was flashed, one by one, followed by all of the circles flashing again. At this point, the participant had to click on the previously flashed circles in the proper sequence. They had 12 s to respond. The test began with sequences of length 4. There were two trials for each sequence length. If the participant was correct on at least one of the two trials, the sequence length was increased for the next trial, up to length 7. The test ended when the participant missed two trials of the same length. The primary outcome measure was the length of sequence the participant could accurately recall before making two consecutive mistakes. This test was modeled after the Corsi Spatial Span test (Corsi, P, 1972; Della Sala et al., 1999).

#### Experimental paradigms

##### Approximate number sense dots test

On each trial, participants were shown an array of blue and yellow dots for 200 ms. There were 5–20 dots of each color, and dot size varied. The participant’s task was to report whether there were more blue dots or more yellow dots. Participants had 10 s to respond. There were 100 trials. The primary outcome measure was accuracy, defined as the proportion of correct responses. For details, see Halberda et al. (2008, 2012).

##### Flanker interference

The Flanker paradigm required participants to indicate the direction of a central arrow flanked by arrows facing either the same direction (congruent) or the opposite direction (incongruent). The flanker arrows were displayed for 100 ms before target onset. The target and flankers were presented together for 50 ms, and then, all arrows disappeared and were replaced by a fixation cross for 70 ms. Participants were instructed to press the “x” key on their keyboard to report a left-pointing target or the “m” key to report a right-pointing target. Participants had three seconds to respond to each trial, and were instructed to respond as quickly as possible. On trials where a participant’s response time exceeded the 85th percentile of their incongruent trial response time distribution, they were warned to go faster. There were 96 trials. The primary outcome measure was the accuracy (proportion of correct trials) difference between congruent trials and incongruent trials. For details, see Passell et al. (2019).

##### Gradual onset continuous performance task (GradCPT)

In this task, the participant sees a series of 300 outdoor scenes, comprised of 90% street scenes and 10% mountain scenes, presented within a circular window. Images were presented for roughly 800 ms, and the transition between images was gradual. Participants were instructed to press a button when they saw a street scene, and to withhold response when they saw a mountain scene (10% of images). The primary outcome variable was *d’*. To compute *d’*, we used the commission error (CE) rate and omission error (OE) rates. The hit rate (*H*) was defined as 1-CE rate, or the proportion of target mountain scenes participants correctly withheld a response to. The false alarm rate (*F*) was the OE rate, or the number of non-target street scenes participants withheld responses to. We used the equation *d’* = *z*(*H*) −* z*(*F*). In the cases where no CEs (*H* = 1.0) or OEs (*F* = 0.0) were made, we used the standard procedure (Macmillan & Creelman, 2005) of deducting or adding one-half error to each measure to prevent *d’* from being undefined. For further methodological details, see Fortenbaugh et al. (2015).

##### Multiple object tracking (MOT)

This paradigm presented participants with an array of 10 identical black disks. At the beginning of each trial, a subset of disks would blink, alternating between a black and a green smiley face for 1000 ms to identify them as targets. All disks would then move randomly around the screen for 5 s. The participant’s task was to track the target disks. At the end of the trial, all disks stopped moving and the participant had 15 s to click on all of the target disks. Correct responses were indicated by green smiley faces, incorrect responses by red frowning faces. There were 3 sets of 6 trials, for a total of 18 trials. The number of targets increased from 3 in the first set to 4 in the second set to 5 in the third set. The primary outcome measure was accuracy, computed as the total number of correct responses divided by the total number of targets tracked. For details, see Passell et al. (2019) or Wilmer et al. (2016).

##### Spatial configuration visual search

This paradigm presented participants with an array of line segments arranged into “T” or “L” configurations. The participant’s task was to search for the target letter “T”, and report whether it was rotated 90° to the left or to the right. The letter “L” served as the distractor, and could appear in any of the four 90° rotations. There were two blocks of trials. In the first block, the total number of items (set size) was 4, and in the second block the set size was 12. Each trial had one target; the remaining items were distractors. Participants had 5 s to respond. There were 100 trials. The primary outcome measure was the slope of the reaction time (for correct trials only) by set size function.

##### Visual working memory (VWM)

In this test, participants were shown a memory array of four novel objects for 1 s. The objects then disappeared. After a 1000-ms retention interval, a single probe object was presented at one of the four locations, and participants were asked to make an unspeeded judgment as to whether or not the probe was the same object that was presented at that location in the original array. There were a total of six novel objects that were randomly sampled throughout the test. There were 4 practice trials and 42 test trials. The primary outcome measure was the number of correct responses (max = 42).

### Data analysis

The data were cleaned and formatted for a series of factor analyses to understand the latent characteristics across the 14 measures. Scores were considered outliers if they were three times the interquartile range below the 25^th^ percentile or above the 75^th^ percentile of the outcome distribution. Participants with outliers on more than one measure were identified and excluded from the factor analysis. We also log-transformed those measures with highly skewed distributions.

While we had a priori expectations about how the experimental tests would relate to one another (all except perhaps GradCPT would load onto an *a* factor), the relationships among the neuropsychological tests and between the two classes of tests were left open. We therefore conducted an initial exploratory factor analysis, followed by a set of confirmatory factor analyses. Since our power analysis indicated at least 350 participants for the exploratory factor analysis, we randomly selected 55% of the participants (training group, *n* = 357) to investigate the underlying factor structure. We held the remaining 45% of the participants (testing group, *n* = 279) for the confirmatory factor analyses. The two groups showed no statistical difference on the performance of the 14 measures and on the demographic characteristics.

#### Exploratory factor analysis

We used several converging methods to determine how many factors to retain, beginning with a parallel analysis. Developed by (Horn, 1965), parallel analysis compares eigenvalues extracted from the analysis data against eigenvalues calculated from randomly generated correlation matrices using the same number of observations and variables. The number of factors to retain is determined by the number of eigenvalues from the analysis data that are larger than those that were randomly generated. The models with various number of factors were then compared in terms of the degree of fit assessed by three goodness-of-fit indices: Tucker–Lewis Index (TLI), root mean square of residuals (RMSR), and root mean square error of approximation (RMSEA), as well as the RMSEA 90% confidence interval (CI). A good fit is defined by TLI > 0.95, RMSR < 0.05, and RMSEA < 0.06 with lower value of CI close to 0 and upper value no more than 0.08 (Browne & Cudeck, 2016; Hu & Bentler, 1999). RMSR or RMSEA < 0.08 indicates an acceptable model fit.

Since all the measures are testing some aspects of cognitive ability, it would not be realistic to assume that any extracted latent structure is truly independent from the others. Therefore, we used maximum likelihood for eigenvalue calculation, factor extraction, and oblimin rotation when extracting more than one factor, to allow the factors to correlate.

#### Confirmatory factor analysis

Confirmatory factor analysis was performed using the held-out testing sample to assess and compare goodness-of-fit between the extracted factor structures and the other candidate structures. Based on prior literature, we hypothesized two factors, the general attention factor (*a*), and a sustained attention factor. We tested whether this hypothesis was supported by the observed data in terms of model fit indices such as comparative fit index (CFI, > 0.90 for acceptable fit, and > 0.95 for good fit) and standardized root mean square residual (SRMR, < 0.05 for good fit, and < 0.08 for acceptable fit, Hu & Bentler, 1999), in addition to TLI and RMSEA. Since the competing factor structures were not nested in nature, we followed the non-nested model comparison sequence as recommended by Merkle et al. (2016). We employed Vuong’s (1989) test to first determine whether the candidate models had equal fit to the same data, or whether the models were distinguishable. If they were distinguishable, we further tested whether one model fit significantly better than another using the non-nested likelihood ratio test (LRT). A final factor structure was distinguishable from the other candidate models and had acceptable model fit in both the exploratory factor analysis and confirmatory factor analysis.

Given the diverse sample and multiple ways to respond, we further assessed measurement invariance in the entire sample (*n* = 636) across demographic groups and response device groups (see Supplemental material in Thornton & Horowitz, 2020) using multigroup confirmatory factor analysis. The measurement invariance testing involves comparing models with increasing constraints. This begins with configural invariance, in which the same factorial structure is fitted to subgroups separately and factor loadings are allowed to vary freely (i.e., unconstrained model). Then, metric invariance (also called weak invariance) is tested by assessing the difference on goodness-of-fit indices of models imposing equality in factor loading across subgroups and the unconstrained models. If metric invariance holds, the next step is to test scalar invariance by further constraining intercepts to be equivalent across subgroups. The measurement invariance is determined by the insignificant changes (Δ) in model fit indices such as ΔCFI (≤ 0.01) and ΔRMSEA (≤ 0.015) (Cheung & Rensvold, 2002), especially ΔCFI which is more robust to sample size than chi-square (Δ*χ*^2^).

Data manipulation and analyses were all conducted using R 3.6.0 (R Core Team, 2020). Exploratory factor analyses were obtained using the *psych* package (v1.8.12, Revelle, 2018), the *lavaan* package (v0.6-5, Rosseel, 2012) for the confirmatory factor analyses, *nonnest2* (0.5-4, Merkle & You, 2020) for the Vuong tests, and *semTools* (v0.5-2, Jorgenson et al., 2019) to test for measurement invariance.

## Results

### Participants

#### Recruitment and retention

**Fig. 1:**
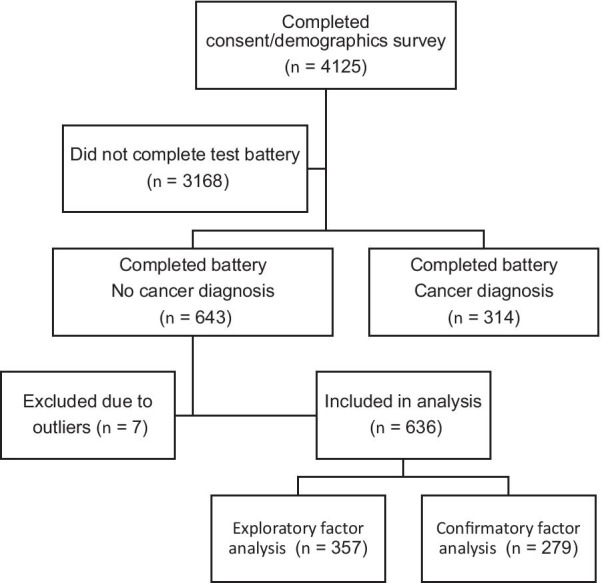
Study participation flow chart

As depicted in Fig. 1, 4125 people completed the consent form and the demographic questionnaire (see below). Of those, 957 ended up completing the test battery, including 643 who reported not having a diagnosis of cancer. The latter group comprised the sample for this study. Seven people were found to have outliers on more than one measure and thus excluded from analysis. As a result, the analysis group contains 636 participants.

#### Demographics

The basic demographic characteristics of the participants are shown in Table 1. Participants were relatively young, but the majority were older than 25. A majority reported female gender. More than half had not finished college. Since the sample was web-based, we do not know participants’ geographic locations. Therefore, we cannot judge whether the racial/ethnic breakdown reflects larger populations (i.e., USA, the continent of North America, etc.). However, the proportion reporting an Asian race was larger than one would expect from a purely US sample.

**Table 1:** Characteristics of participants

Characteristics	Frequency (%) or mean (SD)
Age	30.89 (± 14.33)
18–24	292 (45.9%)
25+	344 (54.1%)
*Gender*	
Male	277 (43.6%)
Female	347 (54.6%)
Missing	12 (1.9%)
*Ethnicity*	
Non-Hispanic	554 (87.1%)
Hispanic	40 (6.3%)
Missing	42 (6.6%)
*Race*	
White	394 (61.9%)
Black	19 (3.0%)
Asian	134 (21.1%)
Other	34 (5.3%)
Missing	55 (8.6%)
*Education*	
High school or less	179 (28.1%)
Some college	177 (27.8%)
College or above	267 (42.0%)
Missing	13 (2.0%)

### Measure performance and correlations

Table 2 summarizes the outcomes used for each measure and their descriptive statistics. The two Trail Making Test tests (TMT-A, TMT-B) showed highly skewed distributions. After log-transformation, the distribution of these two tests was close to normal.

**Table 2:** Descriptive statistics of individual measure performance

	Outcome	Mean	SD	Min	P50	Max	Skewness	Kurtosis	Internal Reliability
*Traditional Clinical neuropsychological tests*									
Letter cancellation	Num. correct	92.247	21.809	8	91	152	0.010	3.108	0.95
Digit symbol coding	n. correct trials	55.047	13.693	16	54	116	0.861	5.371	0.93
Backward digit span	Length of longest sequence	6.102	2.031	1	6	11	0.385	2.747	0.68
Forward digit Span	Length of longest sequence	6.929	1.665	1	7	11	0.252	2.819	0.73
Arithmetic	Points	21.231	6.019	3	22	37	− 0.300	2.730	0.85
Spatial span	Span	5.423	0.970	0	5	7	− 0.720	6.268	0.58
TMT-A	Resp. Time (ms)	32,517.290	18,753.160	12,168	26,579	177,934	2.599	13.347	0.95
Log(TMT-A)		− 10.273	0.456	− 12.089	− 10.188	− 9.407	− 0.792	3.511	
TMT-B	Resp. Time (ms)	47,964.500	26,575.030	14,419	40,647.5	286,388	3.040	19.373	0.96
Log(TMT-B)		− 10.671	0.441	− 12.565	− 10.613	− 9.576	− 0.644	3.754	
*Cognitive Psychology/experimental Paradigms*									
Approximate number sense	Accuracy	0.812	0.055	0.540	0.815	0.930	− 0.596	4.058	0.60
Multiple object tracking	Accuracy	0.822	0.094	0.514	0.833	1.028	− 0.509	2.928	0.92
Visual working memory	Number correct	34.421	4.031	20	35	42	− 0.668	3.334	0.67
Flanker interference	Conflict Resp. time	0.108	0.149	− 0.833	0.083	0.917	0.760	9.334	0.85
Visual search	Search slope (ms/item)	57.450	20.129	− 12.070	55.625	138.180	0.390	3.856	0.75
Gradual onset CPT	d'	2.744	0.808	− 1.356	2.832	4.653	− 0.845	4.942	0.88

**Fig. 2:**
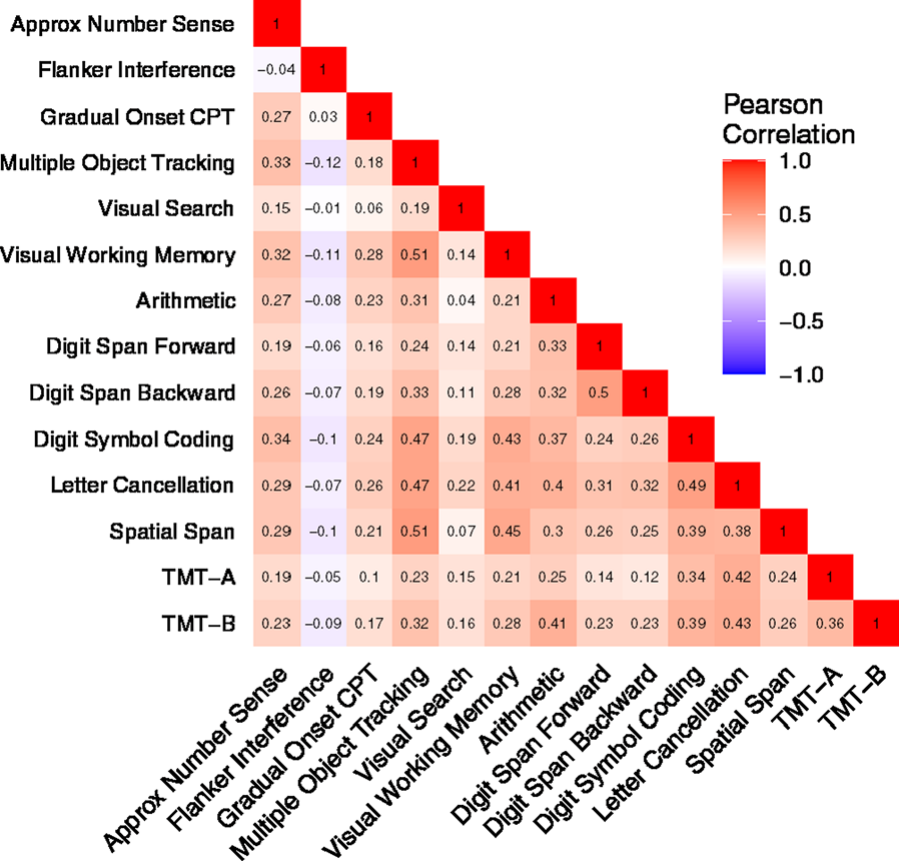
Correlation matrix. Note that TMT-A and TMT-B are log-transformed, and the sign of correlation coefficients was flipped for TMT-A, TMT-B, and Visual Search

Figure 2 presents the correlation matrix of the 14 measures. Since scores for the two Trail Making Test tests and Visual Search were based on time, higher scores indicate poorer performance. In contrast, scores for the remaining measures were accuracy-based, and higher score indicates better performance. Therefore, we flipped the sign of the correlation coefficients for the time-based measures. While the most measures showed positive associations, the Flanker test, when measured by conflict accuracy, was weakly or even negatively correlated with the other tests.

### Exploratory factor analysis

**Fig. 3:**
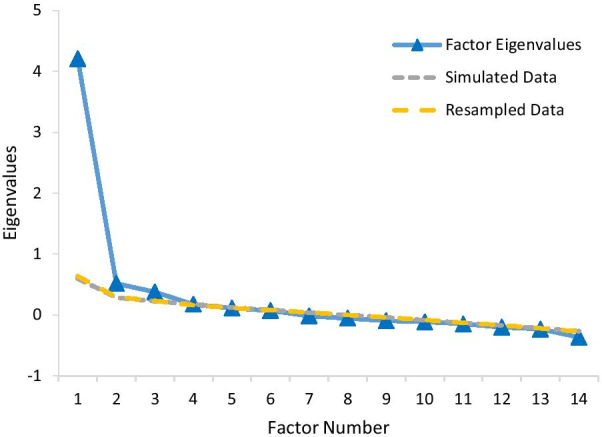
Parallel analysis scree plot

The exploratory factor analysis was conducted using the training group of 357 participants randomly selected from the full sample of 636. The parallel analysis shown in Fig. 3 suggested three factors. The first factor accounted for 34.5% of the total variance, and the first three factors together account for over 50% of the variance.

Next, we extracted one- to five-factor structures to compare goodness of fit. In addition to the three factor structure suggested by parallel analysis, the four- and five-factor structures also achieved good model fits (see Table 3).

**Table 3:** Goodness of fit of the five exploratory factor structures

	TLI	RMSEA (90% CI)	RMSR	BIC	SABIC
One factor	0.836	0.082 (0.071, 0.092)	0.064	− 194.227	50.053
Two factors	0.905	0.063 (0.049, 0.075)	0.046	− 224.492	− 21.454
Three factors	0.979	0.030 (0.000, 0.047)	0.030	− 238.140	− 73.172
Four factors	1.008	0.000 (0.000, 0.029)	0.022	− 204.908	− 74.837
Five factors	1.014	0.000 (0.000, 0.027)	0.017	− 157.549	− 59.203

A good fit is defined by TLI > 0.95, RMSR < 0.05, and RMSEA < 0.06 with lower value of CI close to 0 and upper value no more than 0.08

**Fig. 4:**
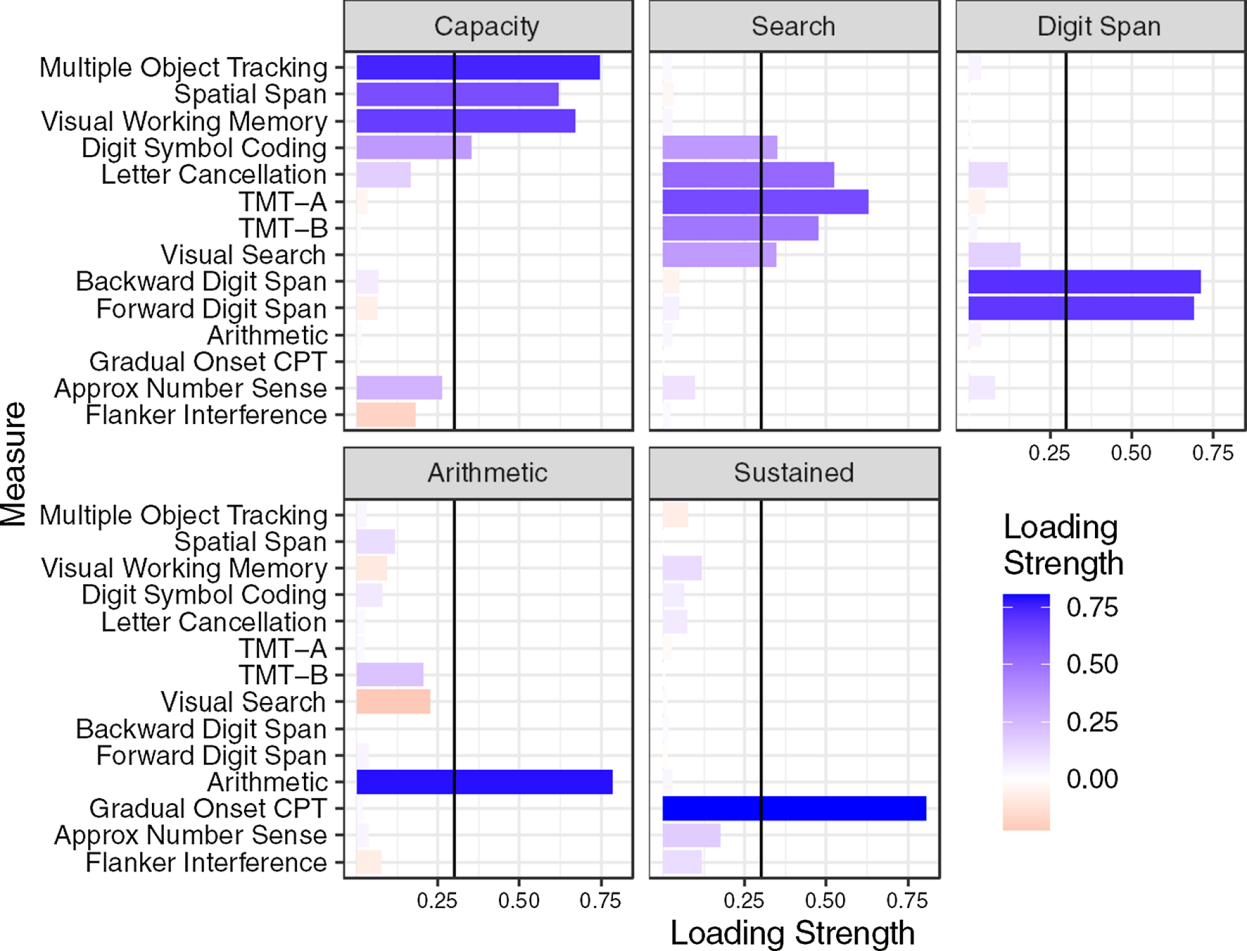
Factor loadings from the five-factor exploratory factor analysis. Blue indicates positive loadings, red negative. The vertical line on each panel denotes the .30 threshold for inclusion of a measure in a given factor

**Fig. 5:**
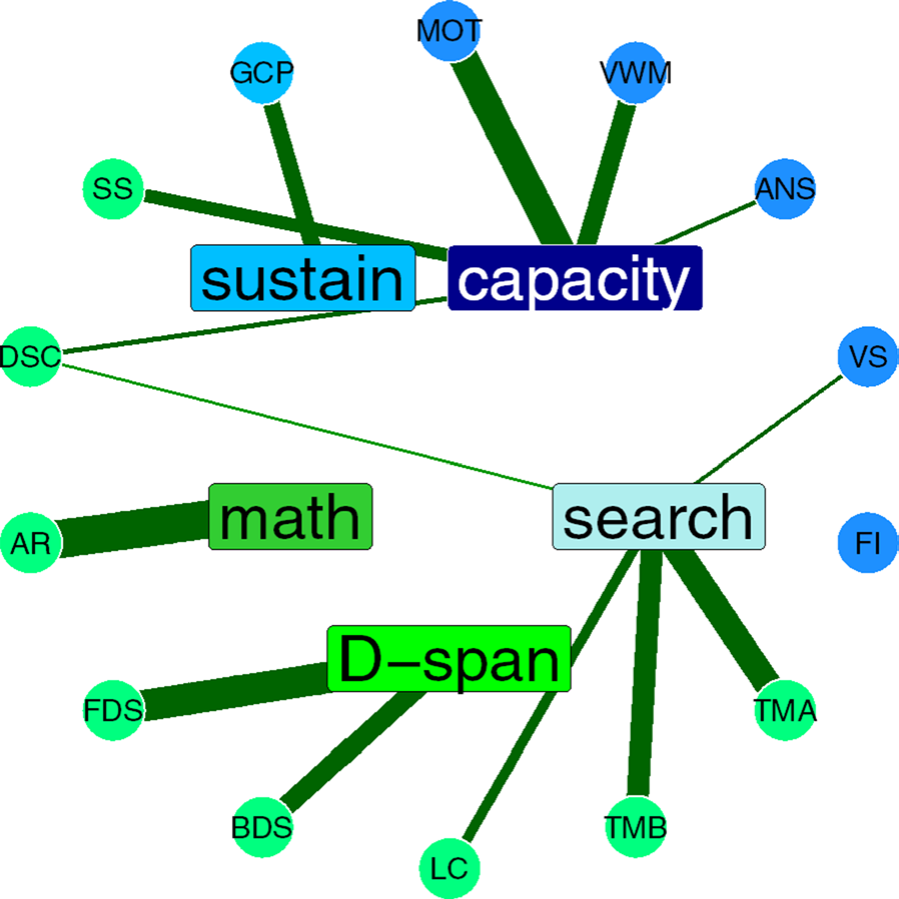
Graphic depiction of five-factor exploratory factor analysis structure. Tests are disks on the outer circle, factors rectangles on the inner circle. Thickness of lines indicates factor loadings, green for positive, red for negative. Darker blue tests were cognitive paradigms predicted to load onto the *a* factor. The light blue test (GCP) was predicted to load onto sustained attention factor. Neuropsychological tests are depicted in green

Figure 4 shows the factor loadings of the five-factor structure, also depicted graphically in Fig. 5. We named the five factors (1) Attentional Capacity, (2) Search, (3) Digit Span, (4) Arithmetic, and (5) Sustained Attention. All but one measure loaded highly on at least one of the five factors, using a minimum factor loading of 0.3. Flanker Inference had low loading on all five factors, which was not surprising given the low correlations observed in the correlation matrix. Although Digit Symbol Coding had a loading over 0.3 on the first (Capacity) factor, its loading onto the second (Search) factor was close to 0.3. Three factors had only one or two measures with high loadings, including the Digit Span factor, the Arithmetic factor, and the Sustained Attention factor (GradCPT only). The factor loadings for the one- to four-factor structures are available in “Appendix B”. In the three-factor structure, GradCPT loaded onto the first factor and Arithmetic loaded onto the second factor, while Visual Search did not load onto any factor. (Its loading on the first factor was 0.246, not meeting out 0.300 criterion.) In the four-factor structure, Arithmetic moved onto its own factor, while Visual Search loaded onto the second factor. Finally, the five-factor structure moved GradCPT onto the Sustained Attention factor. Table 4 shows the factor correlation matrix from the five-factor solution. Next, we used confirmatory factor analysis to compare model fit of the three exploratory factor structures and two other candidate structures.

**Table 4:** Factor correlation matrix from the five-factor exploratory factor analysis

	Capacity	Search	Digit span	Arithmetic	Sustained
Capacity	1				
Search	0.61	1			
Digit span	0.45	0.35	1		
Arithmetic	0.43	0.45	0.40	1	
Sustained	0.44	0.25	0.30	0.29	1

### Confirmatory factor analysis

Due to consistently low factor loadings on all the extracted factors, Flanker was excluded from the confirmatory factor analysis. We assessed the model fit for all factor solutions using the held out testing group of 279 participants. These included a one-factor solution in which all tests would load onto a general attention factor, and a two-factor solution where experimental and neuropsychological paradigms and tests clustered on independent factors, as well as the three, four, and five factor structures derived from the exploratory factor analysis.

Because the model comparisons require all models to be specified on the same set of measures, we kept Visual Search in the three-factor structure even though its highest factor loading was only 0.246. As shown in Table 5, the three structures extracted from the exploratory factor analysis had much better model fit than the two a priori structures. The four-factor structure and the five-factor structure had slightly better model fit than the three-factor structure. The Vuong’s test indicated that the three-factor structure and the four-factor structure were distinguishable (*p* = 0.018), but they fitted the Testing group equally well based on the non-nested LRT (*p* = 0.060). The four-factor structure and the five-factor structure were indistinguishable (*p* = 0.375). The five-factor structure, however, is more theoretically plausible, since sustained and selective attention measures should be independent (Parasuraman et al., 1998).

**Table 5:** Goodness of fit of factor structures

	CFI	TLI	RMSEA (90% CI)	SRMR	*χ*^2^	*df*	AIC	BIC
A priori* structures*								
One Factor	0.871	0.845	0.078 (0.065, 0.092)	0.062	176.387	65	12,774.15	12,868.56
Two Factors	0.881	0.855	0.076 (0.062, 0.090)	0.061	166.579	64	12,766.34	12,864.38
*Structures extracted from the exploratory factor analysis*								
Three factors	0.927	0.908	0.060 (0.045, 0.075)	0.053	124.657	62	12,728.42	12,833.73
Four factors	0.936	0.917	0.057 (0.041, 0.073)	0.050	114.744	60	12,722.51	12,835.08
Five factors	0.938	0.915	0.058 (0.042, 0.074)	0.049	110.770	57	12,724.53	12,847.99
*Hierarchical factor model with five factors*								
	0.932	0.914	0.058 (0.043, 0.074)	0.052	120.742	62	12,724.51	12,829.81

**Fig. 6:**
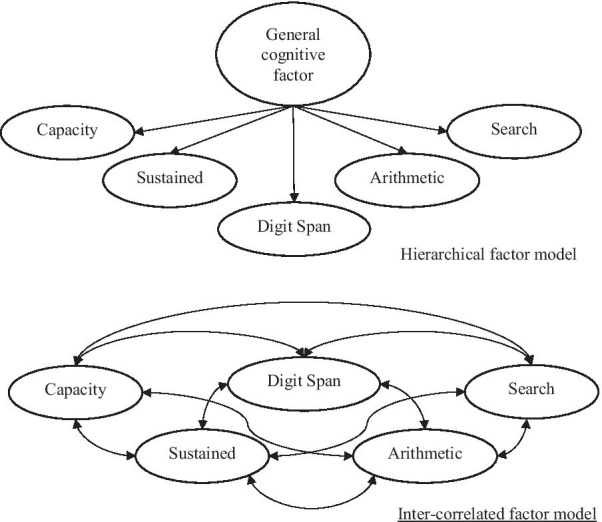
Graphic depiction of hierarchical factor model with five factors and the model with five inter-correlated factors

Given the positive correlations between the five factors derived from the exploratory factor analysis (see Table 4), we further tested a hierarchical factor model (see Fig. 6) where a general factor was imposed above the five factors. The model fit was similar to that of the five-factor solution without the general factor. The Chi-square test also showed no significant difference (*p* = 0.076 for Δ*χ*^2^(Δ*df* = 5) = 9.972) in terms of model fit between the two models. The model comparison result supported the existence of a general cognitive factor. However, the poor model fit of the single-factor structure in both exploratory and confirmatory factor analyses suggested that the five more-specific factors measure unique aspects of cognitive ability. Therefore, the five-factor structure was selected as the final model.

### Measurement invariance

We collected information about the type of device participants used to respond for each measure. There were two general types of response mode, keyboard/mouse click and touchscreen. Participants were allowed to switch response devices between measures. To analyze the possible effect of response mode on the final factor structure, we included only participants who used one device consistently for all measures. There were 535 participants who used one device for all measures. Of these, 418 participants used either keyboard or mouse to respond, while 117 participants used touchscreen only.

The results showed that the metric invariance did not hold between the two mode groups (ΔCFI = 0.01, ΔRMSEA = 0.003, and *p* < 0.01for Δ*χ*^2^(Δ*df* = 8) = 25.694), indicating that individual factor loadings differed as a function of response mode.

Assessments of measurement invariance for demographic factors are reported in “Appendix C”—Measurement invariance analysis by demographic characteristics.

## Discussion

Both cognitive neuroscience and neuropsychology purport to measure cognitive functions, using a largely overlapping terminology (“attention”, executive function”, working memory”, etc.). However, the two fields are largely separate, with different goals and different institutional bases, and we know very little about how well concepts and measures from the two fields overlap. Our goal in this paper is to start making connections between the two fields in order to improve both neuropsychological assessment and our broader scientific understanding of cognition and the brain.

We hypothesized that the five selective experimental attention paradigms (ANS, MOT, VWM, Visual Search, and Flanker Interference) would load onto the *a* general attention factor reported by Huang et al. (2012). We could then use the degree to which the neuropsychological tests loaded onto *a* as an index of how well they function as (selective) attention measures. Neuropsychological tests that did not load on to *a* might load on to a common factor with the GradCPT, suggesting that they measure sustained attention. Or the neuropsychological tests might not be related at all to the experimental paradigms. The results that we actually observed were more complex than the scenarios we envisioned a priori. Our results are more consistent with a five-factor structure that can explain the observed correlations.

### The five-factor structure

We settled on a five-factor structure, based on converging evidence from the scree plot, goodness-of-fit metrics, and theoretical considerations. These comprise: (1) an attentional capacity factor, (2) a search factor; (3) a digit span factor; (4) an arithmetic factor, and (5) a sustained attention factor. Flanker Interference did not load on to any factor.

The first factor comprised three experimental paradigms (MOT, VWM, and ANS), and two neuropsychological tests (Digit Symbol Coding and Spatial Span). Based on the nature of the three experimental paradigms, we tentatively label this the attentional capacity factor.

A second factor comprised the experimental Visual Search paradigm and three neuropsychological tests: Letter Cancellation, TMT-A, and TMT-B. All of the neuropsychological tests have a visual search component: the Letter Cancellation test requires the participant to look for the letter “d” with 2 lines among “d” with one or three lines and the letter “p” with one to three lines. It thus closely resembles a conjunction foraging search task (Jóhannesson et al., 2017; Kristjánsson et al., 2020). Both versions of the Trail Making Test require sequential search for alphanumeric characters. Therefore, we think of this factor as picking up variance related to search or attentional shifting. An important caveat here is that the configural Visual Search paradigm itself loaded less strongly onto this factor than the neuropsychological tests.

One interesting finding is that Digit Symbol Coding loaded almost equally on the first two factors. This is not entirely surprising given that there is a clear search component to the test. Participants need to find the target symbol in the key to find the correct response. Over the course of the test session, the key mappings will become automated and the search component will decrease in importance. If the mappings were to shift from trial to trial, this test would probably load more strongly on the search factor.

The third factor is fairly easy to characterize, as it included both the Forward Digit and Backward Digit Span tests, and nothing else. The Arithmetic test formed the fourth factor. These findings are in line with previous factor analytic studies of neuropsychological tests (Mirsky et al., 1991; Schmidt et al., 1994).

The fifth factor included just the GradCPT. We had predicted that this paradigm would not load onto the same factor as the other experimental cognitive paradigms, since it should measure sustained attention, rather than selective attention, and these faculties are known to be independent (Parasuraman et al., 1998). However, we did expect to see some of the neuropsychological tests to load onto this factor as well, which they did not.

A possible compromise between the single-factor and five-factor structures is the stratified model, where the five specific factors are nested under a general factor. The fit of this model was not statistically distinguishable from the five-factor model in our analyses, so it provides another way to look at the data. We assume that this general factor corresponds to something like the general intelligence *g* factor of the Cattell–Horn–Carroll model, rather than the general attention factor *a* proposed by Huang et al. (2012). In this context, it is worth noting that there was disagreement among the namesakes of the Cattell–Horn–Carroll model as to whether the general stratum was necessary (McGrew, 2009), and the recent work fitting the model to neuropsychological data eschew the general factor in favor of intercorrelated lower-order factors (Agelink van Rentergem et al., 2020; Jewsbury et al., 2017).

### Relationship to other factor analyses of attention tasks

We assumed that our five selective attention paradigms would load on to a single factor, corresponding to Huang et al.’s (Huang et al., 2012) *a*. This was not what we observed. Only three paradigms MOT and VWM (and, if we’re generous, ANS) loaded into the first factor. Visual Search loaded onto the second factor with several of the neuropsychological tests, and Flanker Interference did not load onto any factor. This difference from Huang et al.’s analysis is not due to the fact that we found five factors, while Huang et al. found only one. If we limit ourselves to a single factor, Visual Search and Flanker Interference still do not load onto this factor (though all of the other tests do, see Table 6).

**Table 6:** One-, two-, three-, and four-factor loadings

	One factor	Two factors	Three factors	Four factors
*Model fit*				
TLI	0.836	0.905	0.979	1.008
RMSEA	0.082	0.063	0.030	0.000
	(0.071, 0.092)	(0.049, 0.075)	(0.000, 0.047)	(0.000, 0.029)
RMSR	0.064	0.046	0.030	0.022
BIC	− 194.227	− 224.492	− 238.140	− 204.908
SABIC	50.053	− 21.454	− 73.172	− 74.837

	FA1	FA1	FA2	FA1	FA2	FA3	FA1	FA2	FA3	FA4
*Factor loadings*										
MOT	0.704	0.644	0.096	0.706	0.06	0.013	0.702	0.08	− 0.002	0.011
VWM	0.603	0.54	0.098	0.731	− 0.066	− 0.004	0.743	− 0.017	0.003	− 0.076
S-Span	0.595	0.551	0.068	0.605	0.038	0.009	0.559	0.029	0.042	0.064
DSymbolCoding	0.647	0.681	− 0.044	0.478	0.265	− 0.054	0.441	0.259	− 0.035	0.062
ANS	0.493	0.384	0.173	0.49	− 0.041	0.118	0.486	− 0.073	0.058	0.117
TMT-A	0.561	0.686	− 0.167	0.032	0.686	− 0.094	− 0.001	0.748	− 0.02	− 0.058
TMT-B	0.718	0.753	− 0.023	− 0.022	0.836	0.06	0.035	0.696	0.055	0.138
L-Cancel	0.724	0.715	0.034	0.256	0.484	0.084	0.252	0.446	0.083	0.101
Search	0.235	0.241	0.002	− 0.012	0.246	0.046	− 0.002	0.329	0.12	− 0.171
BDS	0.458	− 0.021	0.827	0.039	− 0.052	0.781	0.189	− 0.092	0.547	0.08
FDS	0.483	0.114	0.631	− 0.031	0.095	0.705	− 0.035	0.03	0.921	− 0.016
Arith	0.589	0.482	0.18	0.127	0.366	0.227	− 0.004	0.014	0.002	0.992
GradCPT	0.343	0.257	0.132	0.377	− 0.084	0.096	0.354	− 0.121	0.065	0.122
Flanker	− 0.094	− 0.058	− 0.06	0.021	− 0.077	− 0.075	0.007	− 0.044	− 0.066	− 0.04

It is important to note that our study was not intended as a replication of Huang et al. (Huang et al., 2012). As we noted in the Introduction, there is no single definitive version of an experimental paradigm. The tests that we employed to instantiate the five paradigms we selected to represent the *a* factor differed in ways large and small from those used in the original Huang et al. study. In the MOT test, whereas our participants tracked 3–5 out of 10 items, Huang et al.’s tracked 4 of 8. Huang et al. measured VWM using a full-report technique, whereas we used a single-item probe technique. Our Visual Search paradigm was a search for a rotated T target among rotated Ls. Huang et al.’s Configuration Search paradigm was a search for a square composed of a white rectangle above a black rectangle among squares with the opposite spatial configuration. Instead of the even/odd judgment used in Huang et al.’s Counting test, our ANS test requires participants to judge which of two sets visible on the screen is larger. The dependent measure for Counting was a reaction time slope, as opposed to accuracy for the ANS. Furthermore, Huang et al.’s Counting task spanned the subitizing (3–4) and estimation ranges (13–14) ranges, while the ANS samples only the estimation range. As we noted in the Introduction, the Flanker Interference task is substantially different from Huang et al.’s Response Selection test.

Furthermore, factor analysis is sensitive to the context of the battery. Our battery included only four of the nine “primary” paradigms and none of the nine “secondary” paradigms used in Huang et al.’s battery. We also included the GradCPT and eight neuropsychological tests that were not in Huang et al.’s battery. This certainly affected the factor structure.

In contrast to Huang et al. (2012)’s single factor solution, Skogsberg et al. (2015) obtained a four-factor structure for their battery: Global Attention, Sustained Attention, Transient Attention, and Spatiotemporal attention. Unfortunately, there are only two tasks in common between their battery and ours. Their Central Focusing Task corresponds to Flanker Interference, and both batteries included MOT. Furthermore, the reliability of the Central Focusing task was too low for it to be included in the analysis.

In the Skogsberg et al. (2015) data, MOT forms part of the Spatiotemporal Attention factor, so it is tempting to identify that with our Attentional Capacity factor. However, while MOT and the Spatial Span task fit that description, it is more difficult to see how VWM and Digit Symbol Coding can be thought of as spatiotemporal tasks. Furthermore, the ANS, which weakly loads onto our Capacity factor, would seem to correspond more closely to Skogsberg et al.’s Global Attention factor, since it requires the observer to segregate by color across the visual field. Meanwhile, Skogsberg et al.’s Spatiotemporal Attention factor includes the Spatial Shifting task, which we would predict should load onto with our Attentional Shifting Factor. Thus, our factor structure does not neatly align with Skogsberg et al.’s, although both analyses agree on the existence of a Sustained Attention Factor.

Similarly, it is difficult to map Huang et al.’s (2012) general factor to one of the four factors in Skogsberg et al. (2015). Again, the only paradigm in common between the two datasets is MOT, which would identify Huang et al.’s *a* with Skogsberg’s Spatiotemporal Attention factor, yet many of the paradigms in *a* do not fit that description (e.g., Visual Short-Term Memory, Response Selection, Counting). Perhaps it is our verbal descriptions of the factors that are misleading us here. It would be an interesting project, beyond the scope of this paper, to take the correlation matrices from these three studies (our study; Huang et al., 2012; and Skogsberg et al., 2015), subject them to the same factoring or clustering rules, and attempt some sort of synthesis. Ultimately, however, we are going to need more such studies, ideally using overlapping batteries. The existence of only three factor analytic studies of attention, with only one paradigm in common, points to the severe neglect of individual difference work in this field.

We also think it important to consider the demographic and cultural differences between the three samples. Both of the previous studies used convenience samples of undergraduate students. Huang et al. (2012) studied 257 students aged 17–22 at South China Normal University in Guangzhou, Guangdong, China. Skogsberg et al. (2015) studied 222 students, aged 18–26, from Northwestern University in Evanston, Illinois, USA. No demographic data were provided for participants in either study, though by definition they all possessed some college education.

Our study, in contrast, recruited a global internet sample of 636 people. Our participants ranged in age from 18 to 81, with a mean age of 31. More than half of our sample was older than the participants in the undergraduate studies. We also had much more variation in educational level, with 28% of our sample reporting a high school education or less, and 42% reported having completed a college degree. Finally, while we do not know which countries our participants lived in, only 21.1% reported Asian ethnic background, while 61.1% reported white or European ethnic background. Overall, we have good reasons to believe that there was a lot more demographic heterogeneity in our sample than in the two undergraduate samples.

Demographic characteristics seem likely to influence not only performance but also the observed factor structure. Our measurement invariance analysis (see “Appendix C”—Measurement invariance analysis by demographic characteristics) showed that metric invariance held for age, gender, and education, indicating that factor loadings did not significantly vary as a function of these characteristics. Nevertheless, we suggest that the greater diversity of our sample contributed to differences between the factor structure we observed and those obtained by previous studies, possibly via other characteristics that we did not consider here. Cultural variables may also influence the observed factor structures. Cross-cultural studies have indicated cultural differences in attention and perception between participants of East Asian and Western descent (Amer et al., 2017; Masuda & Nisbett, 2001). All of these issues need to be taken into account when comparing across studies or attempting theoretical generalizations. Now that remote testing platforms have become more widespread, future factor analytic studies should aim to cast a wider net, in order to increase both generalizability and variability.

### Relationship to the Cattell–Horn–Carroll model of cognitive abilities

As we have mentioned, the Cattell–Horn–Carroll model (McGrew, 2009) is an influential model of human cognitive abilities, based in factor analysis. It arose out the field of intelligence measurement, and has recently been shown to fit well to neuropsychological batteries (Agelink van Rentergem et al., 2020; Jewsbury et al., 2017). The Cattell–Horn–Carroll model therefore provides a theoretically and empirically sound approach to classifying neuropsychological tests.

The relationship between Cattell–Horn–Carroll and the way cognition is thought of in cognitive psychology and cognitive science is not clear. Consider attention, the focus of this paper. Cattell–Horn–Carroll is a hierarchy of abilities, with narrow abilities (e.g., “quantitative reasoning”) organized under broad abilities (e.g., “fluid reasoning), with a general cognitive ability stratum at the top (McGrew & Schneider, 2018). There is no “broad ability” corresponding to attention in the Cattell–Horn–Carroll model. Attention is mentioned in many places in the hierarchy under fluid reasoning, working memory capacity, and processing speed ability. In this view, attention is not a single function or ability, but a property of many different subsystems. Jewsbury et al. (2017) proposed a similar view of executive function. Reconciling this approach with the view of attention as an independent factor or factor on its own will require cross-disciplinary collaboration.

### Implications for theories of attention

Our analysis suggests three subcomponents of attention (i.e., attention capacity, search, and sustained attention). For the attention capacity factor, it is not surprising the experimental paradigms of MOT, VWM, and ANS paradigms comprised this factor. The relationship between visual working memory and multiple object tracking has been explored in some depth. It is important to keep in mind that spatial memory and visual working memory are distinct constructs (Klauer & Zhao, 2004; Oh & Kim, 2004; Vicari et al., 2003; Woodman & Luck, 2004). While the most intuitive model of multiple object tracking would involve storing the locations of targets in spatial memory, then moving attention in turn as quickly as possible to each target to update its location, this class of model is unable to account for MOT performance (Pylyshyn & Storm, 1988; Vul et al., 2009), leading theorists to propose additional cognitive structures or operations such as visual indexes (or “FINSTS” – “FINgers of INTsantiation”, Pylyshyn, 1989, 2001) or multifocal attention (Cavanagh & Alvarez, 2005). There is also some dispute as to whether spatial working memory is involved. Allen, et al. (2006) argued that MOT performance was closely linked to Baddeley and Hitch’s spatial working memory store, aka the visuo-spatial sketchpad (Baddeley & Hitch, 1974). However, several studies have shown dissociations between MOT and spatial working memory (Bettencourt et al., 2011; Carter et al., 2005; O’Hearn et al., 2010). Furthermore, Trick et al. (2012) showed that visuospatial ability (including Corsi Blocks, a spatial span variant) but not working memory, predicts MOT.

The most striking link between the two paradigms is that they seem to have a similar capacity limit of around four items (Cowan, 2001). Vul et al. (2009) used an ideal observer model to show that the limit on the number of objects that can be tracked is not a property of the information available in the paradigm and therefore must derive from a limitation in either memory or attention. This analysis does not conclusively link MOT and VWM, but it does raise the possibility that their common variance might derive from reliance on a common attentional resource. Fougnie and Marois (2006) explicitly posited that the common capacity limit in the two paradigms (as well as rapid serial visual presentation paradigms) derived from a common reliance on visuospatial attention. However, Souza and Oberauer (2017) argued that VWM and MOT use different attentional resources.

Electrophysiological studies also demonstrate close links between VWM and MOT. The contralateral delay activity, a sustained voltage decrease during the retention interval of a short-term memory test, indexes the number of items held in visual working memory (Luck & Vogel, 1997; Luria et al., 2016). The amplitude of this activity can also be used to measure the number of targets being tracked in an MOT experiment (Drew & Vogel, 2008; Drew et al., 2011, 2012). This suggests an overlap in the neural circuitry involved in the two paradigms.

While the relationship between ANS, on the one hand, and VWM and MOT, on the other, is not well studied, it is worth considering the theoretical relationship between enumeration and attention. Numerosity is just one of a set of summary or ensemble statistics that can be extracted by the visual system (Alvarez, 2011). There is some evidence these representations are derived independently from one another (Khvostov & Utochkin, 2019; Utochkin & Vostrikov, 2017). However, there may be some core faculty for computing statistics that is held in common, and is also useful for tracking and remembering objects. Alternatively, it may be that there is something unique about numerosity or magnitude perception that makes it a probe for attention.

Meanwhile, what does it mean that Visual Search did not load onto the first factor? Visual search has long been identified as a core paradigm in the modern study of attention, dating back to Treisman and Gelade’s seminal work (1980), yet here it does not load with other commonly used attentional paradigms. These results may be telling us something about the fractionation of attention. Performing a difficult spatial-configuration search with little guiding information will rely on directing focal attention to each item in turn (Moran et al., 2016; Woodman & Luck, 1999), and therefore much of the variance may be due primarily to variations in the speed with which participants can shift attention. A paradigm like the ANS, on the other hand, requires a global distribution of attention across the display, with no shifting. Similarly, most accounts of MOT assume that attention is continuously distributed to all items in parallel, rather than shifting from one target to another in turn (Howe et al., 2010; Pylyshyn & Storm, 1988), unless target identities also must be tracked (Oksama & Hyönä, 2008). Attention also seems to be required for maintenance in visual working memory (Balestrieri et al., 2019; Heuer & Schubö, 2016). In some cases, this involves discrete shifts of spatial attention (Williams et al., 2013); it is not clear if it is also possible to maintain attention to multiple items in parallel, as in MOT.

### Implications for interpreting data from clinical research studies

The major impetus behind this project was our survey of meta-analyses of the neuropsychological research on cancer-related cognitive impairment (Horowitz et al., 2019). One of our findings was that there was a great deal of variability in how tests were assigned to domains. For example, the Digit Symbol Coding was classified as a test of processing speed 43.1% of the time, as an attention test 32.3% of the time, and as an executive function test 24.6% of the time. Furthermore, many tests classified as attention tests, such as Digit Span, seemed to us to have little face validity. This project was conceived as a way to provide some empirical guidance for what should be classified as an attention test and what should not, an approach that we hope will be adopted for other domains as well.

One conclusion from this study is that, in line with our initial impressions, Digit Span and Arithmetic tests should not be classified as attention tests. This is not a novel finding. Mirsky et al. (1991) conducted a factor analysis of putative neuropsychological attention tests, and found that Digit Span and Arithmetic tests did not load onto the same factor as Trail Making, Digit Symbol Coding, Letter Cancellation, Stroop and Continuous Performance tests. Digit Span and Arithmetic are probably best thought of as Working Memory tests, as specified in the Wechsler Adult Intelligence Scale standard model (Reynolds et al., 2013). Agelink ven Rentergen et al.’s (2020) factor analysis of neuropsychological tests also found that in the best-fitting model the Digit Span tests formed their own “working memory” factor.

On the positive side, we found evidence Digit Symbol Coding and Spatial Span do seem to be tapping attentional capacity, while Trail Making and Letter Cancellation measure attentional shifting. These five tests could continue to be classified as attention tests, on the basis of these results, though reports should distinguish between Capacity and Search ability.

### Implications for future clinical research studies

The connection between a subset of the neuropsychological tests and the cognitive attention paradigms is a two-way street. Not only does this finding validate that the neuropsychological tests have some connection to the construct of attention, it also suggests that certain experimental paradigms might be usefully adapted to serve clinical purposes.

The standard armamentarium of clinical neuropsychology has a number of limitations (Marcopulos & Łojek, 2019), including lack of sensitivity, lack of process-purity (Kessels, 2019), and lack of repeatability (Bilder & Reise, 2019). Developing tests from cognitive neuroscience paradigms, which tend to be theoretically derived to be more process-pure, and designed for repeatability, is a potential solution (Kessels, 2019). Whether such tests would be more sensitive is an empirical question.

Experimental cognitive paradigms do have drawbacks as potential clinical tests (Kessels, 2019). Their psychometric properties, including sensitivity and reliability, are generally not known. Most paradigms have been tested primarily on college undergraduates, meaning not only is their generalizability in question, but also that without extensive norming studies, there is no way to adjust an individual’s score for factors like age, sex, and education. Determining clinical utility will require normative data with clinical and nonclinical samples. Many paradigms rely on response time, and may become unreliable when different populations adopt different speed-accuracy tradeoffs. Since each study adapts the paradigm to answer a specific question, there are innumerable variants of each paradigm, so there is no standard to adopt. And while they are typically not proprietary, by the same token they cannot simply be used off the shelf; some investment is necessary to produce a useful version.

We do not mean to minimize these problems. However, we do think that they can be overcome. The Cognitive Neuroscience Treatment Research to Improve Cognition in Schizophrenia initiative, for example, has been leveraging cognitive psychology and cognitive neuroscience paradigms to develop tests to improve our understanding of schizophrenia (Barch et al., 2009; Carter & Barch, 2007), and a similar initiative is underway for cognitive deficits associated with obesity and diabetes (d’Ardenne et al., 2019).

Our findings suggest that if a condition leads to deficits on the Digit Symbol Coding or Spatial Span tests, then a test based on MOT, for example, might be useful. Tullo et al. (2018) have begun developing MOT as a clinical test of attentional capacity. Similarly, if deficits are observed using the Trail Making Test, it might be worth using a visual search paradigm (Gold et al., 2007; Horowitz et al., 2006) to determine whether the problem stems specifically from a problem in shifting attention, or whether it might be attributable to the other faculties tapped by the Trail Making Test.

### Limitations and caveats

There are a number of important limitations to this study. First, we are sampling from the population of people who are online and self-selected to participate in cognitive studies for free. As noted above, we think that our sample is probably more representative than, say, undergraduate university psychology students. However, it is better-educated, younger, and probably more affluent than the population as a whole. The majority of subjects were recruited through TestMyBrain.org. In past studies using TestMyBrain.org, the top sources for the site have been www.google.com, www.stumbleupon.com, and www.i-am-bored.com, and frequently used search terms leading to TestMyBrain.org were “brain test” and “brain tests”, suggesting that many visitors arrive at the website because they are curious about their cognitive abilities (Germine et al., 2012). Furthermore, we do not have a good idea of which countries our participants live in, and our demographic information does not line up with standard US racial/ethnic categories. Critically, we do not know how the factor structure might change for specific clinical populations. We are currently studying a population of cancer survivors, but interested researchers might want to carry out replications in their fields of interest.

Second, our analysis noted significant effects of response mode on the factor structure; whether participants used a keyboard and mouse or a touchscreen made a noticeable difference. We did not have enough participants who used only a touchscreen to fully characterize this effect, but since computing as a whole, and computerized neuropsychological testing in particular, is moving toward touchscreen interfaces, this issue will become increasingly important.

Perhaps the most important limitation of our study is that our neuropsychological tests were not necessarily identical to the tests currently being administered by clinical neuropsychologists. A challenge of the present study was converting traditional paper-and-pencil tests to an online format while keeping the differences between the two to a minimum. Instructions for TestMyBrain measures were given visually, and practices were completed in order to ensure comprehension of instructions. In contrast, a neuropsychologist administers the pencil-and-paper versions and instructions are given orally. The traditional Arithmetic and Digit Span tests require participants to verbally answer, the Trail Making Test, Digit Symbol Coding, and Letter Cancellation necessitate the use of a pen or pencil, and Spatial Span requires finger pointing. Our online measures were modified so that participants could respond using either a keyboard or touchscreen. In “Appendix A”—Comparison of online and traditional, we detail the traditional pencil-paper tests and their modified online counterparts. In addition to administration and formatting differences, digitizing pencil-and-pencil tests may alter the perceptional, cognitive and motor performances of tests and introduce measurement bias due to device variations (Germine et al., 2019).

## Conclusions

The goal of this project is to provide a bridge between theory-driven cognitive research and clinically relevant neuropsychological research. We believe it is important to align neuropsychology with cognitive psychology and cognitive neuroscience to improve the precision and interpretability of cognitive assessments. Our results should provide guidance for which neuropsychological tests should be classified as attention tests, and hopefully provide inspiration for the development of new clinical assessments based on experimental attention paradigms. Furthermore, we hope we have provided a template for other researchers to explore the connections between cognitive paradigms and neuropsychological tests in domains beyond attention. By bringing these fields closer together, we can improve our scientific understanding of cognition, and ultimately improve the welfare of people who suffer from cognitive disorders and deficits.

## Data Availability

The datasets generated and/or analyzed during the current study are available in the Open Science Foundation repository, https://osf.io/py83d/.

## Appendix A: Comparison of online and traditional tests

TMT-A & TMT-B were similar between the online and paper and pencil version except for the implement used for connecting circles. In our online TMT-A & TMT-B, participants had to connect a series of circles on their device screen, in ascending order. For Form A, 25 circles contained numbers and had to be connected in ascending numerical order (e.g., 1-2-3) by drawing a line between circles. For Form B, 25 circles contained letters and numbers, and had to be connected in ascending numerical and alphabetical order, alternating between numbers and letters (e.g., 1-A-2-B-3-C). Depending on the device (i.e., laptop/desktop or tablet/smartphone), participants could use either their mouse or their finger to connect the circles. In the traditional Trail Making Test, participants are given a pencil and are asked to connect the circles. The primary outcome variable for both versions is the time it takes to connect all the circles, calculated separately for Parts A and B.

In the online Digit Symbol Coding test, participants had to choose which number matched a target symbol shown on screen, using a given symbol-number key. Participants could use either a keyboard or touchscreen. Participants in the paper and pencil version are given a pencil and a sheet of paper with the key located at the top of the page and rows of numbers. Participant are tasked to copy the symbol below each number. The primary measure for both online and original versions was how many matches the participant correctly makes in 90 s.

The online and paper and pencil Letter Cancellation tests are comparable in asking participants to search an array of “d” and “p” letters for instances of the lowercase letter “d” flanked by various arrangements of 2 lines. Anytime the participant saw the target letter, they were then asked to cross the letter out (by clicking or touching the letter for the online test or striking through the target letter with a pencil for the original version) until all instances of the letter were found or 20 s passed. Participants’ online score was the total number of correctly identified letters. Various calculations are derived from the traditional letter cancellation test but most notably, the sum of the number of target letters (Bates & Lemay, 2004).

The Arithmetic test required participants to solve a series of arithmetic computation and word problems of increasing difficulty. The online version was modeled after the arithmetic test of the Wechsler Adult Intelligence Scales III and was presented visually. For each online question, the participant could earn 1 point for a correct answer and 2 points for a correct answer given within 10 s, regardless of the time allowed or difficulty of the trial. The primary outcome variable was total points earned across the test. There was a total of 20 possible questions. Time limits were given for each question, 15 s for the first 5 questions, then 30 s for the next 5 questions, 60 s for the following 9 questions, and 120 s for the final question. For the traditional test, participants are orally presented with a series of word problems and are not supplied with a pencil or paper. Participants are timed beginning after each problem is read and participants must respond orally within a time limit. Time limits are 15 s for the first 6 questions, then 30 s for 7–11 questions, 60 s for 12 -19 questions, and 120 s for question 20. If the participant gets 4 consecutive wrong answers, then the test is stopped. For questions 1–18, 1 point is obtained for a correct answer given within the time limit; and for questions 9- 20, 2 points are obtained if a correct answer was given within 10 s or 1 point if answer was given within the time limit.

Both online and traditional digit span tests required participants to recall strings of digits of increasing length. The forward digit span test required participants to recall digits in the order they were presented. The backward digit span test required them to recall the digits in reverse order. The online tests were adapted from the Digit Span tests of the Wechsler Adult Intelligence Scales. For the online version, digit sequences are presented visually, and participants are asked to memorize the numbers and then either keyboard or finger press the digits. There are two trials for each sequence length presented and the test ends when the participant misses two trials of the same length. The longest possible sequence length is 11 digits. Participants have 4 s to respond before they are warned to keep responding with the remaining number of digits. Score for the digit span test is calculated as the highest number of digits participants were able to successfully recall at least once—in other words, the length of the longest sequence where participants got at least one of two trials right. For the traditional version, participants are orally presented the digit sequences and asked to verbally recite the sequence. The test ends when the two trials for the same sequence length is incorrect or when the maximal sequence length is reached (9 digits forward, 8 backward). Each correct response is worth one point.

In the Spatial Span test, participants had to learn and recall sequences of visually presented spatial locations. For the online test, sequences were indicated by a shape that changed color. When clicking on each dot in a sequence, participants have 12 s to click on the next dot. If at any point they take more than 12 s to click the next dot, they timeout and the next trial begins. The sequences increased in length from 4 to 7. The primary outcome measure was the length of sequence the participant could accurately recall before making two consecutive mistakes. For the traditional Spatial Span test, the administrator taps the spatial sequence on a board that contains an array of 10 blocks. Participants are then asked to reproduce the sequence by tapping the blocks in the same order they were presented. The test comprises eight sequence lengths, from 2 to 9, with two trials for each sequence length (Brown, 2016).

## Appendix B: Factor loadings from the exploratory factor analysis

See Tables 6 and 7.

**Table 7:** Factor loadings for the five-factor solution

	FA1	FA2	FA3	FA4	FA5
Multiple object tracking	0.805	0.022	0.009	0.021	− 0.064
Visual working memory	0.614	0.030	0.023	− 0.078	0.141
Spatial span	0.506	0.047	0.041	0.066	0.076
Digit symbol coding	0.348	0.293	− 0.038	0.058	0.137
Approx number sense	0.324	− 0.018	0.065	0.096	0.234
TMT-A	− 0.031	0.768	− 0.030	− 0.059	0.038
TMT-B	0.073	0.676	0.066	0.148	− 0.063
Letter cancellation	0.205	0.458	0.092	0.099	0.059
Visual search	0.007	0.321	0.124	− 0.172	− 0.008
Backward digit span	0.158	− 0.100	0.619	0.062	− 0.011
Forward digit span	− 0.054	0.048	0.859	− 0.019	0.016
Arithmetic	− 0.008	0.010	0.003	0.991	0.012
Gradual onset CPT	0.009	− 0.006	0.040	0.063	0.566
Flanker interference	− 0.096	0.000	− 0.078	− 0.055	0.152

## Appendix C: Measurement invariance analysis by demographic characteristics

We assessed measurement invariance on three demographic characteristics. In addition to age group and gender, we further collapsed education into two subgroups, “Less than college” and “College and above” so each group had over 250 participants.

**Table 8:** Change in goodness-of-it indices for selected demographic characteristics

	CFI	ΔCFI	RMSEA	ΔRMSEA	*χ*^2^	Δ*χ*^2^	*df*	Δ*df*	*p*
*Age*									
Configural	0.947	NA	0.055	NA	222.05		114		
Metric	0.943	0.004	0.055	0	238.9	16.841	122	8	0.032
Scalar	0.931	0.012	0.058	0.003	270.41	31.51	130	8	< .001
*Gender*									
Configural	0.961	NA	0.047	NA	193.55		114		
Metric	0.957	0.004	0.048	0.001	209.43	15.882	122	8	0.044
Scalar	0.941	0.016	0.055	0.007	250.68	41.249	130	8	< .001
*Education*									
Configural	0.946	NA	0.054	NA	215.75		114		
Metric	0.944	0.003	0.053	0.001	228.72	12.971	122	8	0.113
Scalar	0.938	0.006	0.054	0.001	247.4	18.675	130	8	0.017

As shown in Table 8, the results supported metric invariance for all three characteristics where the changes in fit indices were smaller than the recommended cutoff values for CFI and RMSEA as defined in the Confirmatory Factor Analysis subsection of the Data Analysis section. The scalar invariance held for education only in terms of ΔCFI and ΔRMSEA.

## Task abbreviations

ANS: Approximate number sense
GradCPT: Gradual onset continuous performance task
MOT: Multiple object tracking
TMT-A: Trail making test, version A
TMT-B: Trail making test, version B
VWM: Visual working memory

## Statistical abbreviations

CFI: Comparative fit index
LRT: Likelihood ratio test
RMSR: Root mean square of residuals
RMSEA: Root mean square error of approximation
TLI: Tucker–Lewis Index
